# A Bio-Inspired Framework for Reducing Appearance Bias Dominance and Framing Sensitivity in Chest X-Ray Classification

**DOI:** 10.3390/biomimetics11080595

**Published:** 2026-08-20

**Authors:** Ganbayar Batchuluun, Sung Jae Lee, Su Jin Im, Kang Ryoung Park

**Affiliations:** Division of Electronics and Electrical Engineering, Dongguk University, 30 Pildong-ro 1gil, Jung-gu, Seoul 04620, Republic of Korea; ganabata87@dongguk.edu (G.B.); sungje0319@dgu.ac.kr (S.J.L.); sujin125917@dgu.ac.kr (S.J.I.)

**Keywords:** bio-inspired learning, chest X-ray classification, explainable artificial intelligence, appearance bias dominance, reliable deep learning

## Abstract

Although deep learning methods have shown high performance in chest X-ray classification, high accuracy alone does not guarantee reliable reasoning. A model may still exhibit pathological behavior, such as unstable evidence usage under harmless input changes, inconsistent reasoning across augmented views, excessive dependence on surrounding frame information, and appearance bias dominance, where prediction relies too heavily on intensity while neglecting texture and shape. In this paper, we propose a bio-inspired pathology-aware, factor-aware framework for explainable and reliable chest X-ray classification, inspired by biological vision principles such as figure–ground separation, selective attention, and balanced use of complementary visual cues. During training, the method regularizes appearance bias dominance through evidence-guided counterfactual perturbations that mimic cue-suppression analysis in biological perception, thereby revealing and penalizing excessive factor dependence. During testing, it evaluates model behavior using four criteria: reasoning stability, augmentation inconsistency, appearance bias dominance, and framing sensitivity. This combination enables the framework to go beyond conventional inference-time explanation by both correcting pathological behavior during training and exposing it during evaluation. From a biomimetic perspective, the framework encourages the model to separate relevant foreground anatomy from surrounding background and to avoid over-reliance on a single dominant cue. The proposed approach improves interpretability and reliability without modifying the backbone architecture or increasing model size or inference-time cost. The proposed training process improved the F1-score of DenseNet-121 from 0.899 to 0.931, while also producing more stable and balanced reasoning.

## 1. Introduction

Chest X-ray classification is one of the most important applications of deep learning in medical imaging because chest X-rays are fast, inexpensive, and widely used in clinical practice. Although modern deep networks can achieve high classification accuracy, correct prediction does not necessarily mean correct reasoning. A model may still rely on unstable, incomplete, or pathological evidence. Two central problems motivate this work. The first is appearance bias dominance, where the model becomes overly dependent on one visual factor while underusing others. For example, it may become overly dependent on shape and ignore texture and pixel intensity, or rely too strongly on intensity while underusing shape and texture. The second is framing sensitivity, where the model relies excessively on contextual framing or background features instead of the relevant foreground information. Both behaviors are dangerous because a model may still predict the right class while reasoning for the wrong reasons. In contrast, bio-inspired visual processing emphasizes foreground-relevant information, suppression of irrelevant background context, and balanced use of multiple visual cues rather than excessive dependence on a single cue. Existing explainability methods, such as class activation map (CAM)-based visualization [1], are useful for showing where the model attends, but they are usually used only after training. They can reveal problematic behavior, yet they do not directly correct it during optimization. As a result, a model may remain biased, unstable, factor-dominated, or frame-sensitive even when its explanation map looks reasonable. This creates a gap between post hoc interpretation and direct control of model behavior.

To address this gap, we propose a bio-inspired pathology-aware, factor-aware framework for chest X-ray classification, motivated by biological vision mechanisms that emphasize figure–ground separation, selective attention, and the integration of complementary visual cues. During training, the method uses CAM for attention correction in a bio-inspired manner, encouraging the classifier to separate foreground anatomical evidence from irrelevant frame and background information. If the extracted heatmap focuses more on the frame or background than on the foreground object region, the model is further optimized with object-area guidance so that its attention is pushed back toward the relevant evidence. In parallel, appearance bias dominance is handled through mask-guided factor perturbations rather than CAM, reflecting a bio-inspired strategy in which the importance of different visual cues is examined by selectively weakening them. Specifically, shape dependence is examined by tile shuffling within the mask region, texture dependence is examined by blurring within the same region, and pixel-intensity dependence is examined by reducing the masked-region intensity by 50%, which weakens intensity without removing the underlying shape or texture pattern. In this way, the model can be penalized when it becomes overly dependent on only one factor.

During testing, the framework evaluates model behavior using four criteria: reasoning stability, augmentation inconsistency, appearance bias dominance, and framing sensitivity. These criteria examine whether the model preserves the same evidence under harmless changes, whether reasoning shifts across different views of the same image, whether one appearance factor dominates excessively, and whether the decision relies too strongly on surrounding frame information.

Inspired by biological vision, the framework strengthens figure–ground separation, promotes object-focused attention, and encourages balanced use of complementary cues, guiding the classifier toward anatomically meaningful features instead of irrelevant background regions or one overly influential cue. The proposed framework therefore goes beyond conventional inference-time explanation by combining training-time behavioral correction with test-time pathological analysis. Importantly, this is achieved without changing the backbone architecture or increasing model size or inference-time cost. The result is a more stable, balanced, and interpretable chest X-ray classifier that aims not only to be accurate, but also to reason in a more trustworthy way.

Our Main Contributions:

The main contributions of this work are as follows:

- We propose a pathology-aware framework for chest X-ray classification that addresses two central reasoning problems, appearance bias dominance and framing sensitivity. During training, the method corrects pathological attention drift by using CAM to detect when the model focuses more on frame or background than on the foreground object region, and it then guides the model back toward the object area.
- We introduce a mask-guided factor regularization strategy to handle appearance bias dominance, where the model becomes overly dependent on one factor while underusing others. Specifically, tile shuffling inside the mask is used to disrupt shape, blurring inside the mask is used to weaken texture, and reducing masked-region intensity by 50% is used to test intensity dependence without removing the underlying shape or texture pattern.
- We propose a test-phase behavioral evaluation framework based on four criteria, reasoning stability, augmentation inconsistency, appearance bias dominance, and framing sensitivity, allowing the model to be assessed not only by classification accuracy, but also by how consistently and reliably it reasons.
- We made the source code for the proposed method publicly available on GitHub [2].

## 2. Related Work

### 2.1. X-Ray Image Classification Foundations

Large public datasets made modern chest X-ray classification possible. ChestX-ray8 introduced frontal-view chest radiographs from patients with text-mined disease labels and also included a weakly supervised classification/localization benchmark [3]. CheXNet then showed that a 121-layer DenseNet could achieve strong pneumonia detection performance on ChestX-ray14 and extended the setting to all 14 findings [4]. Chest eXpert (CheXpert) further expanded the scale of chest radiograph learning by releasing a large number of radiographs from patients together with uncertainty labels and expert comparison sets, and it has since become one of the standard benchmarks for chest X-ray classification [5].

### 2.2. Post Hoc Explanation and Localized Interpretation

For visual explanation, CAM showed that class-discriminative localization can be obtained by combining global average pooling with class-specific weights, making it possible to identify discriminative regions even when training uses only image-level labels [1]. Grad-CAM broadened the applicability of this idea across diverse CNN architectures by using gradients from the last convolutional layer to produce coarse localization maps without altering or retraining the network in medical imaging [6]. These methods are widely used because they are simple and practical, but they are primarily post hoc tools: they help inspect model attention after prediction rather than directly enforcing better reasoning during training.

Recent chest X-ray-specific work has pushed explanation beyond plain heatmaps. Confidence-Aware Probabilistic Class Activation Map (CAPCAM) is a weakly supervised framework for detecting and localizing multiple thoracic conditions in chest X-rays. It integrates the CX-Ultranet backbone, probabilistic CAM pooling, a Confidence-Aware Network (CAN), and an Anomaly Detection Network (ADN), while enabling localization without bounding-box annotations during training; its source code is also publicly available [7]. Chest X-Ray Explainer (ChEX) is not a standard classifier baseline but a broader chest X-ray explainer: it supports textual prompts or box queries, predicts grounded region descriptions, and is evaluated across nine localized chest X-ray tasks; the authors also released official code [8].

### 2.3. Shortcut Learning, Confounders, and Behavior-Aware Supervision

A major concern in trustworthy medical imaging is that high accuracy can hide the use of the wrong cues. Shortcut learning was formalized by Geirhos et al. as the tendency of deep networks to rely on decision rules that work on benchmark data but fail under changed conditions [9]. In chest radiography, the study showed that pneumonia detection performance can vary substantially across hospital systems, demonstrating weak cross-hospital generalization [10]. DeGrave et al. later showed that COVID-19 chest-radiograph models could exploit confounding factors rather than true pathology, meaning that apparently strong performance could still be driven by shortcuts [11].

Several recent chest X-ray methods try to move beyond pure post hoc explanation. Chest X-ray Image Classification: A Causal Perspective constructs a structural causal model and uses backdoor adjustment with tailored probability optimization to reduce confounding effects in CXR classification; the authors provide official code [12]. Gaze-guided graph neural network (GazeGNN) incorporates raw radiologist eye-gaze and image information into a unified graph representation for chest X-ray classification, instead of first converting gaze traces into visual attention maps, and official code is also available [13]. These works are relevant because they try to improve what the model learns, not just how its outputs are visualized.

### 2.4. Adversarial Learning for Alignment of Generated Outputs

Generative adversarial networks (GANs) employ a discriminator to distinguish real samples from generated ones, while a generator is trained to produce outputs capable of deceiving the discriminator. This adversarial learning principle has been extended beyond image synthesis to tasks in which the generated output is a structured representation rather than a natural image. Following this approach, previous work treated CAM heatmaps as generated localization maps and trained a discriminator to distinguish them from ground-truth masks, thereby encouraging the heatmaps to exhibit a mask-like structure [14]. The resulting attention guidance directed the model toward the target region while reducing its reliance on surrounding contextual cues. Because mask generation is commonly addressed using segmentation architectures such as U-Net, prior mask information can also provide a meaningful structural prior for guiding model attention.

Unlike that research [14], our work is closest in spirit to behavior-aware chest X-ray learning, but it differs from the studies above in two ways. First, it uses CAM during training only for frame/background attention correction, rather than as a general-purpose explanation mechanism. Second, it uses mask-guided factor perturbations to regularize appearance bias dominance, specifically shape, texture, and pixel-intensity dependence, while the test phase separately measures reasoning stability, augmentation inconsistency, appearance bias dominance, and framing sensitivity. In this sense, the method is neither merely a post hoc explanation approach nor simply a new classifier backbone, but a training-and-evaluation framework for reasoning behavior. In addition, the proposed foreground-attention mechanism does not require a discriminator network during training, thereby reducing the computational cost and processing time of the training phase.

## 3. Proposed Methodology

### 3.1. Problem Formulation and Research Objective

The proposed framework improves chest X-ray classification by addressing two pathological reasoning problems during learning and evaluation: appearance bias dominance and framing sensitivity. During training, the model is corrected if its CAM heatmap focuses more on frame or background than on the foreground object region. At the same time, the model is evaluated using mask-guided factor perturbations to reveal whether it is overly dependent on one factor, shape, texture, or pixel intensity, while ignoring the others. During testing, the trained model is further analyzed using four behavioral criteria: reasoning stability, augmentation inconsistency, appearance bias dominance, and framing sensitivity.

**Figure 1:**
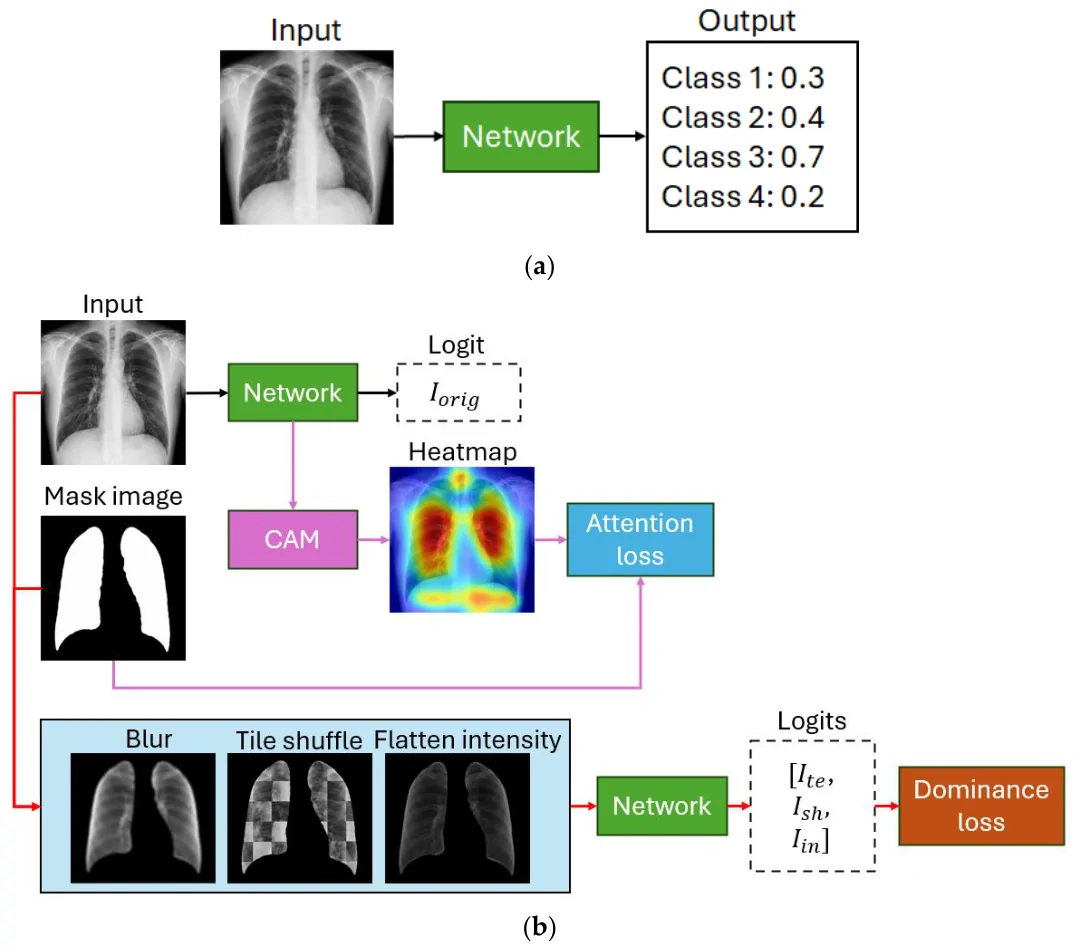
Flowchart of the proposed training method. (**a**) Testing phase; (**b**) training phase.

As shown in Figure 1, the proposed framework consists of both training and testing phases. In Figure 1a, the testing phase is illustrated. Any existing backbone model can be used directly without adding extra tools or modifying its original structure. Therefore, the deployed model keeps the same architecture and inference-time processing cost, while the retrained version is expected to achieve better reasoning behavior and improved performance.

Figure 1b shows the training phase. Two inputs are required: an input chest X-ray image and its corresponding lung mask. The black arrows indicate the first stage, in which the original chest X-ray image is used to train the classifier. The plum-colored arrows indicate the second stage, in which a heatmap is extracted from the trained model using CAM, and the mask image is compared with this heatmap to determine whether the model’s attention is focused on the lung region. Based on this comparison, an attention loss is computed to update cases in which the model focuses more on frame or background than on the lung region.

The red arrows indicate the third stage, which is used to analyze appearance bias dominance. First, the input X-ray image is masked using the lung mask. Within this masked region, three perturbations are applied: blurring (Gaussian blur, kernel size = 11 × 11), tile shuffling (random shuffle on a 4 × 4 grid, i.e., 16 tiles), and 50% intensity reduction. These operations produce three factor-specific images corresponding to weakened texture, disturbed shape, and weakened pixel intensity, respectively. The three images are then fed into the network, and their logits are compared with the original logit obtained in the first stage. Separate losses are computed from these comparisons. If one perturbation causes a much larger logit change than the others, that factor is regarded as dominant in the decision-making process.

Finally, the model is trained again using both the attention correction loss and the dominance loss. Through this process, the proposed framework aims to reduce appearance bias dominance and discourage pathological attention drift toward frame or background, thereby improving the reliability of chest X-ray classification. Algorithm 1 describes the steps of the proposed training method in pseudocode.
**Algorithm 1.** Proposed training processI: input chest X-ray image M: lung mask image y: ground-truth class label f(⋅;θ): classifier model with parameters θ $\lambda_{cam}$: weight of the CAM attention loss $\lambda_{dom}$: weight of the dominance loss1:**Stage 1** Original prediction$y_{t}^{orig}\leftarrow f(I)$2:Classification loss$L_{cls}\leftarrow CE(y_{t}^{orig},y)$3:**Stage 2** CAM extractionH←CAM(I)4:Foreground attention$A_{fg}\leftarrow \sum (H⊙M)$5:Background attention$A_{bg}\leftarrow \sum (H⊙(1-M))$6:Attention loss$L_{cam}\leftarrow max(0,A_{bg}-A_{fg})$7:**Stage 3** Masked image$I_{ma}\leftarrow I⊙M$8:Texture perturbation$I_{te}\leftarrow Blur(I_{ma})$9:Shape perturbation$I_{sh}\leftarrow TileShuffle(I_{ma})$10:Intensity perturbation$I_{in}\leftarrow 0.5\times I_{ma}$11:Texture prediction$y_{t}^{te}\leftarrow f(I_{te})$12:Shape prediction$y_{t}^{sh}\leftarrow f(I_{sh})$13:Intensity prediction$y_{t}^{in}\leftarrow f(I_{in})$14:Logit drops$\Delta_{te}\leftarrow y_{t}^{orig}-y_{t}^{te}$, $\Delta_{sh}\leftarrow y_{t}^{orig}-y_{t}^{sh}$, $\Delta_{in}\leftarrow y_{t}^{orig}-y_{t}^{in}$15:Mean drop$\bar{\Delta }\leftarrow (\Delta_{te}+\Delta_{sh}+\Delta_{in})/3$16:Dominance loss$L_{dom}\leftarrow (\Delta_{te}-\bar{\Delta }{)}^{2}+(\Delta_{sh}-\bar{\Delta }{)}^{2}+(\Delta_{in}-\bar{\Delta }{)}^{2}$17:**Final stage** Total loss$L_{total}\leftarrow L_{cls}+\lambda_{cam}L_{cam}+\lambda_{dom}L_{dom}$18:Parameter update$\theta \leftarrow \theta -\eta \nabla_{\theta }L_{total}$19:Outputf(⋅;θ)

### 3.2. Dataset Description

This study employed the COVID-19 Radiography Database from Kaggle [15], which provides chest X-ray images together with their corresponding lung masks. The dataset comprises 21,165 PNG images distributed across four classes: 10,192 Normal, 6012 Lung Opacity, 3616 COVID-19, and 1345 Viral Pneumonia. Each image has a spatial resolution of 299 × 299 pixels. The COVID-19 Radiography Database is a multi-source collection comprising chest X-ray images obtained from multiple hospitals and datasets across different countries.

The masks, originally generated using a modified U-Net, were used during training to define the relevant anatomical region and guide CAM-based attention. They were not required during inference.

Each class directory contained paired image and mask folders, with corresponding files matched by filename. To address class imbalance, each class was adjusted to 5000 samples. Excess images were removed from larger classes, while smaller classes were expanded using horizontal flipping, ±20° rotation, brightness adjustment, and blurring. This produced a balanced dataset of 20,000 images.

**Figure 2:**
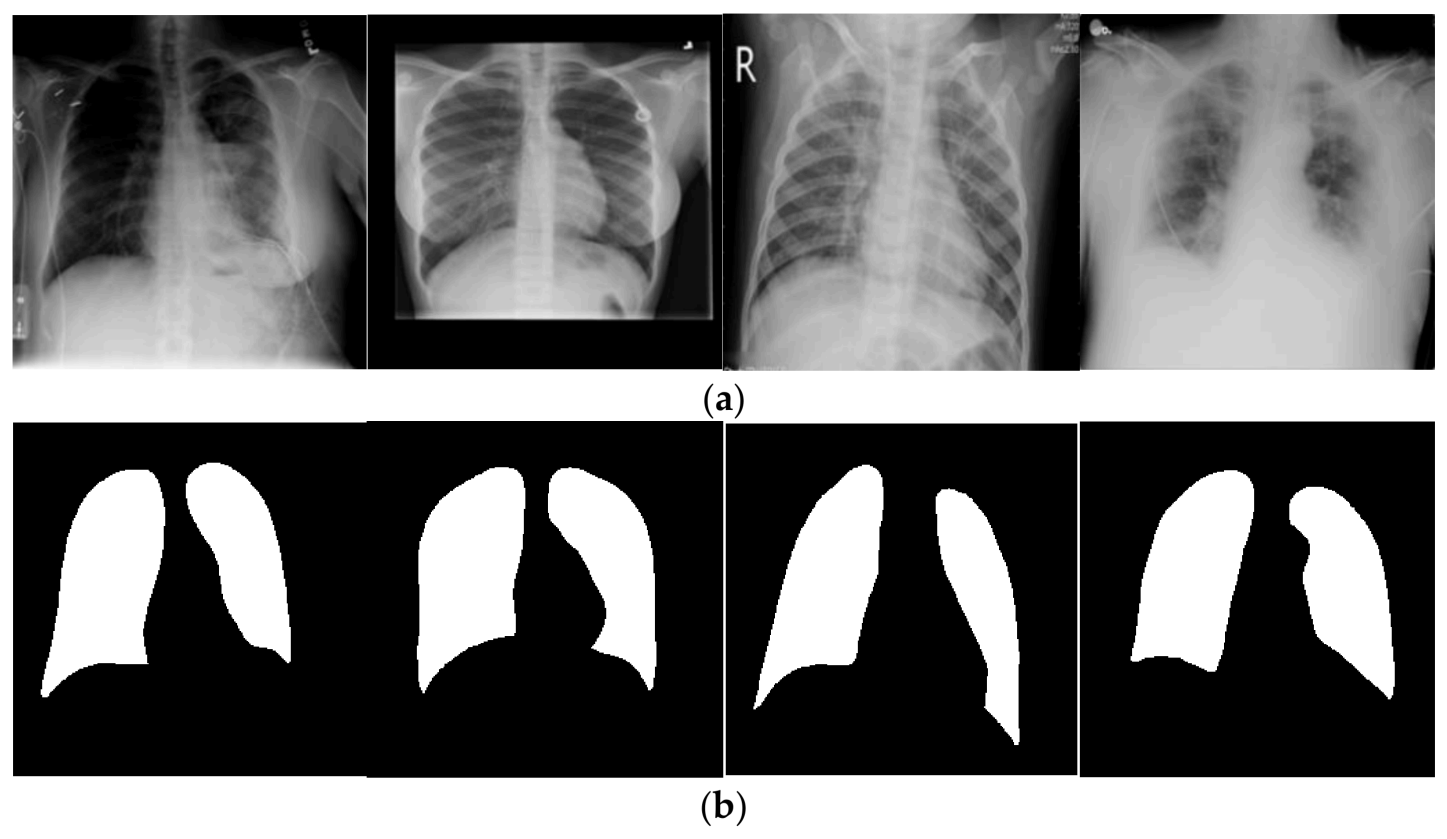
Representative images [15]. The columns show Normal, COVID-19, Viral Pneumonia, and Lung Opacity from left to right. (**a**) X-ray images; (**b**) corresponding lung masks.

A reverse two-fold evaluation protocol was applied. The 20,000 images were partitioned into two equally sized subsets. In each fold, one subset served as the training set, whereas the other was split into 1000 validation images and 9000 test images. The subset roles were reversed in the second fold. Validation data were used only to monitor training, whereas test data were reserved for final evaluation. Figure 2 presents example chest X-ray images and their corresponding mask images.

## 4. Experimental Results

### 4.1. Training Setup

This subsection details the software and hardware environment, selected hyperparameters, and training protocol adopted for the experiments. Table 1 summarizes the computational platform and software specifications, whereas Table 2 provides the hyperparameter values and training settings used for the proposed framework.

### 4.2. Loss Functions

This subsection presents the loss functions used to optimize the proposed framework. The overall training objective comprises three terms: classification loss, Class Activation Map (CAM) attention loss, and dominance loss. The classification term learns the target category from the original chest X-ray image, the CAM attention term penalizes activation outside the lung region, and the dominance term limits excessive reliance on any single visual factor.

- Classification loss

The classification loss is computed from the prediction of the original chest X-ray image:

(1) $$L_{cls}=CE(y_{t}^{orig},y)$$

where CE(⋅) denotes the cross-entropy loss, $y_{t}^{orig}$ is the predicted output of the classifier for the original input image I, and y is the ground-truth class label.

- CAM attention loss

To reduce pathological attention drift, a class activation map H is extracted from the original image branch and compared with the lung mask M. The foreground attention and background attention are defined as:

(2) $$A_{fg}=\sum (H⊙M)$$

(3) $$A_{bg}=\sum (H⊙(1-M))$$

where H is the CAM heatmap, M is the binary lung mask, 1−M represents the non-lung region, and ⊙ denotes element-wise multiplication. The CAM attention loss is then defined as:

(4) $$L_{cam}=max(0,A_{bg}-A_{fg})$$

where max(0,⋅) discourages the model only when the attention outside the lung region is larger than the attention inside the lung region.

- Dominance loss

To analyze appearance bias dominance, three mask-guided perturbations are generated from the masked image region: blurring to weaken texture, tile shuffling to disturb shape, and 50% intensity reduction for pixel intensity. Their corresponding target outputs are denoted as $y_{t}^{te}$, $y_{t}^{sh}$, and $y_{t}^{in}$, respectively. The target output of the original image is denoted as $y_{t}^{orig}$.

The logit drops caused by the three perturbations are defined as:

(5) $$\Delta_{te}=y_{t}^{orig}-y_{t}^{te}$$

(6) $$\Delta_{sh}=y_{t}^{orig}-y_{t}^{sh}$$

(7) $$\Delta_{in}=y_{t}^{orig}-y_{t}^{in}$$

where $\Delta_{te}$, $\Delta_{sh}$, and $\Delta_{in}$ represent the changes in the target output after weakening texture, disturbing shape, and reducing pixel intensity, respectively. Their mean drop is given by:

(8) $$\bar{\Delta }=\frac{\Delta_{te}+\Delta_{sh}+\Delta_{in}}{3}$$

The dominance loss is then defined as:

(9) $$L_{dom}=(\Delta_{te}-\bar{\Delta }{)}^{2}+(\Delta_{sh}-\bar{\Delta }{)}^{2}+(\Delta_{in}-\bar{\Delta }{)}^{2}$$

This loss becomes large when one factor produces a much greater output change than the others, indicating dominant dependence on that factor.

- Total loss

The final training objective combines the three losses as:

(10) $$L_{total}=L_{cls}+\lambda_{cam}L_{cam}+\lambda_{dom}L_{dom}$$

where $L_{total}$ is the final loss used for optimization, $\lambda_{cam}=0.5$ is the weighting coefficient for the CAM attention loss, and $\lambda_{dom}=0.5$ is the weighting coefficient for the dominance loss. These two coefficients control the relative contributions of attention correction and factor-dominance regularization during training.

**Figure 3:**
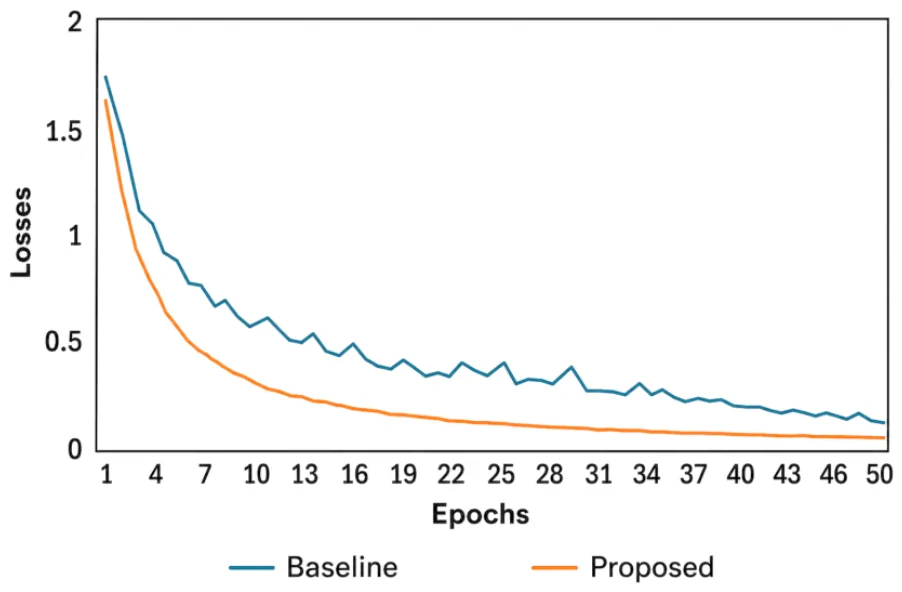
Comparison of DenseNet-121 classification-loss curves under the original and proposed training procedures.

We selected DenseNet-121 as a representative example to illustrate the loss behavior across training epochs, as presented in Figure 3. Figure 3 contrasts the classification-loss curves obtained from standard DenseNet-121 training with those produced when DenseNet-121 is optimized using the proposed framework. The proposed approach shows more stable convergence and maintains lower loss values throughout training than the original procedure.

### 4.3. Testing

#### 4.3.1. Evaluation Metrics

The experiments were evaluated using conventional classification measures together with the newly introduced criteria. Standard classification performance was quantified using the F1-score, derived from the true positive rate (TPR) and positive predictive value (PPV).

(11) $$PPV=\frac{\#TP}{\#TP+\#FP}$$

(12) $$TPR=\frac{\#TP}{\#TP+\#FN}$$

(13) $$F1=\frac{2\cdot PPV\cdot TPR}{PPV+TPR}$$

In these equations, #FP, #FN, and #TP correspond to the counts of false positive, false negative, and true positive predictions, respectively.

The proposed test-phase measurements are reasoning stability, augmentation inconsistency, appearance bias dominance, and framing sensitivity. Unlike conventional classification metrics, these measurements are designed to examine whether the model relies on consistent, balanced, and object-focused evidence.

Reasoning stability measures whether the model preserves similar evidence under small harmless transformations, such as slight rotation, cropping, brightness adjustment, and noise. Let H be the heatmap of the original image and $H^{\prime }$ the heatmap of the transformed image. The reasoning stability score is defined as:

(14) $$RS=1-\frac{\sum |H-H^{\prime }|}{\sum H+\epsilon }$$

where RS denotes reasoning stability, H is the original heatmap, $H^{\prime }$ is the heatmap after harmless transformation, ∣⋅∣ denotes absolute difference, ∑ indicates summation over all pixels, and $\epsilon =10^{-6}$ is a small constant to avoid division by zero. A higher value indicates more stable reasoning.

Augmentation inconsistency measures how much the model’s output changes when different augmented views of the same image are used. Let $y_{t}^{orig}$ be the target output of the original image and $y_{t}^{aug}$ the target output of the augmented image. It is defined as:

(15) $$AI=1-\;|y_{t}^{orig}-y_{t}^{aug}|$$

where AI denotes augmentation inconsistency, $y_{t}^{orig}$ is the target output of the original image, and $y_{t}^{aug}$ is the target output of the augmented version. A lower value indicates more consistent behavior.

Appearance bias dominance measures whether one factor, texture, shape, or pixel intensity, affects the model much more strongly than the others. Let $\Delta_{te}$, $\Delta_{sh}$, and $\Delta_{in}$ denote the target-output drops caused by texture, shape, and intensity perturbations, respectively:

(16) $$\Delta_{te}=y_{t}^{orig}-y_{t}^{te}$$

(17) $$\Delta_{sh}=y_{t}^{orig}-y_{t}^{sh}$$

(18) $$\Delta_{in}=y_{t}^{orig}-y_{t}^{in}$$

Their mean is:

(19) $$\bar{\Delta }=\frac{\Delta_{te}+\Delta_{sh}+\Delta_{in}}{3}$$

Then the appearance bias dominance score is computed as:

(20) $$ABD=1-((\Delta_{te}-\bar{\Delta }{)}^{2}+(\Delta_{sh}-\bar{\Delta }{)}^{2}+(\Delta_{in}-\bar{\Delta }{)}^{2})$$

where ABD denotes appearance bias dominance, $y_{t}^{orig}$ is the target output of the original image, $y_{t}^{te}$, $y_{t}^{sh}$, and $y_{t}^{in}$ are the target outputs after texture, shape, and intensity perturbations, respectively. A larger value indicates stronger dominance of one factor.

Framing sensitivity measures how much the model’s attention depends on the surrounding frame or background rather than the lung region. Let H be the heatmap and M the lung mask. The attention inside and outside the lung region are defined as:

(21) $$A_{fg}=\sum (H⊙M)$$

(22) $$A_{bg}=\sum (H⊙(1-M))$$

The framing sensitivity score is then defined as:

(23) $$FS=1-\frac{A_{bg}}{A_{fg}+A_{bg}+\epsilon }$$

where FS denotes framing sensitivity, $A_{fg}$ is the foreground attention inside the lung region, $A_{bg}$ is the background attention outside the lung region, and ⊙ denotes element-wise multiplication. A lower value indicates less sensitivity to the surrounding frame.

#### 4.3.2. Ablation Study

In this subsection, we compare a baseline model optimized solely with the standard classification loss against a model trained using the proposed CAM-based attention correction and factor-dominance regularization. Three backbone architectures—MobileNet-v1 [22], EfficientNet-B0 [23], and DenseNet-121 [24]—were assessed under both baseline and proposed settings using the same data partitions and training procedures. This design isolates the contribution of the proposed framework while preserving the original backbone architecture.

The 50% reduction is not selected as a performance-tuning hyperparameter. Instead, it is used as a diagnostic perturbation to substantially weaken the pixel-intensity cue while preserving the underlying shape and texture information, allowing the model’s dependence on intensity to be evaluated through the resulting change in output.

For each backbone, evaluation is carried out using five metrics: the F1-score (F1), reasoning stability (RS), augmentation inconsistency (AI), appearance bias dominance (ABD), and framing sensitivity (FS). This allows the ablation study to assess both standard classification performance and the quality of the model’s reasoning in terms of stability, consistency, factor balance, and dependence on surrounding frame information. Higher values indicate better performance for *F1*, *RS*, *AI*, *ABD*, and *FS*.

**Figure 4:**
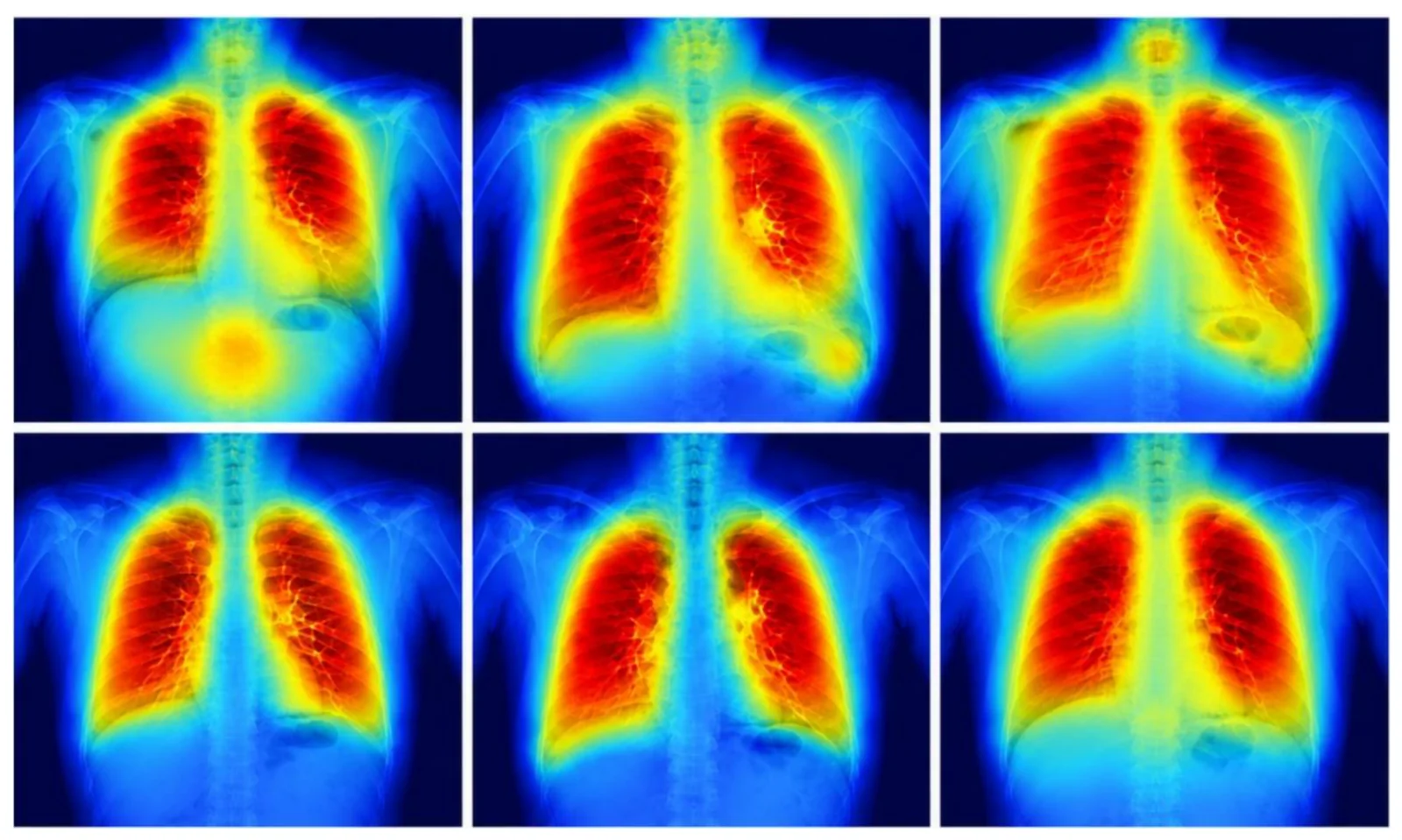
Comparison of heatmaps for the methods presented in Table 3. From left to right, the columns represent MobileNet-v1 [22], EfficientNet-B0 [23], and DenseNet-121 [24], while the top and bottom rows correspond to the baseline and proposed models, respectively.

Table 3 presents the baseline and proposed results for each backbone architecture. Overall, the proposed framework strengthens reasoning quality and often improves classification performance while preserving the original network architecture. Figure 4 further contrasts the heatmaps generated under the two training settings. The baseline models often activate regions outside the lungs, including surrounding structures and background areas, whereas the proposed training strategy produces more lung-focused heatmaps with reduced activation in irrelevant regions. All results reported in this section are test results obtained after training the models with either the baseline or proposed training method. The reported values are the average results of the two-fold evaluation.

As shown in Figure 4, the proposed loss does not force attention away from all extra-lung regions. It is applied only when background attention exceeds foreground lung attention, thereby reducing excessive framing dependence rather than eliminating contextual information. In other words, we do not suppress frame or background attention to zero; instead, we encourage the model to focus more on the lung region and less on the background, while background information can still contribute to the final decision.

Table 4 presents DenseNet-121 as a representative example and compares the confusion matrices obtained from the baseline model and the model trained with the proposed framework using the same test split. The baseline matrix illustrates the original class-wise misclassification patterns, whereas the proposed matrix shows how these patterns change after applying the new training strategy. Table 5 additionally provides class-wise results for positive predictive value, true positive rate, and F1-score.

The results reported in Table 3, Table 4 and Table 5 indicate that the proposed training method achieves better classification performance than the baseline setting.

#### 4.3.3. Comparisons with State-of-the-Art (SOTA) Methods

To examine the generalizability of the proposed training framework, we compared it with several recent chest X-ray-based SOTA methods reported in the literature, including ResNet18-SVM [25], Attention-CNN [26], ViT-ResNet Fusion [27], Fine-tuned Xception-XAI [28], Transfer-Learning Framework [29], and Effective COVID-19 CNN [30]. For fairness, the original architectures of these methods were kept unchanged, and the proposed framework was applied as an additional training strategy. Each method was initially evaluated in its standard baseline configuration and was then retrained with the proposed training process while retaining the same backbone and network structure. This design allows the effectiveness of the attention-guided framework in promoting evidence-centered learning to be examined across multiple architectures rather than within a single backbone alone.

Table 6 summarizes the comparison results. Overall, the proposed training framework improves reasoning behavior and attention localization, and in many cases also enhances classification performance, while preserving the original model architectures.

**Figure 5:**
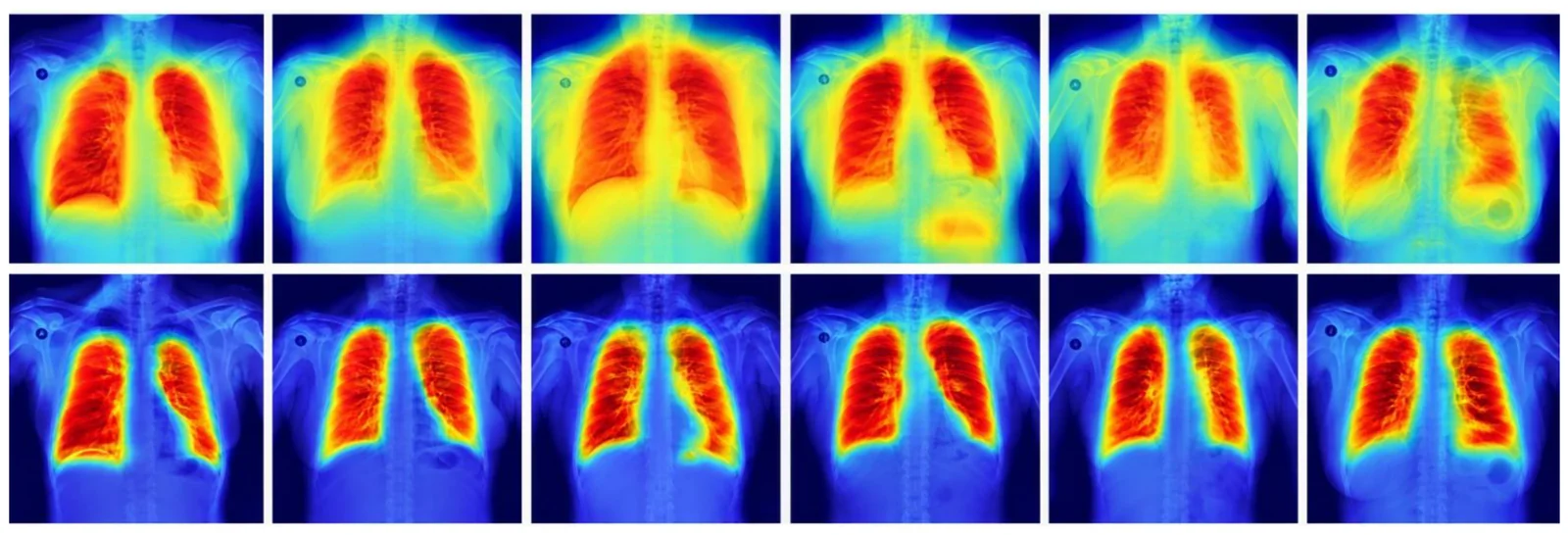
Heatmap comparison of the methods reported in Table 6. From left to right, the columns correspond to ResNet18-SVM [25], Attention-CNN [26], ViT-ResNet Fusion [27], Fine-tuned Xception-XAI [28], Transfer-Learning Framework [29], and Effective COVID-19 CNN [30]. From top to bottom, the rows represent the baseline and proposed methods.

Figure 5 contrasts the heatmaps generated by the baseline and proposed variants of the methods reported in Table 6. The comparison indicates that the proposed framework directs model attention more consistently toward the lung region while reducing appearance bias dominance and framing sensitivity.

#### 4.3.4. Algorithm Complexity and Processing Time

This subsection evaluates the computational efficiency of the proposed framework and baseline methods on the desktop system in Table 1 and the Jetson TX2 platform [31]. The Jetson TX2 features a 256-core NVIDIA Pascal™ GPU, 8 GB of shared memory, and 59.7 GB/s memory bandwidth. Table 7 reports inference time, frames per second (fps), GFLOPs, and parameter counts, confirming that the proposed framework adds no inference-time or parameter overhead.

#### 4.3.5. Statistical Significance and Effect Size Analysis

**Figure 6:**
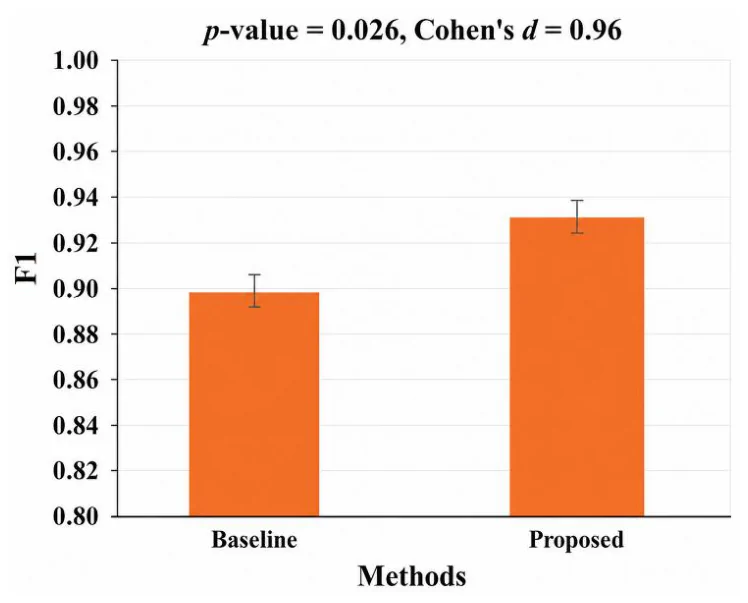
Student’s *t*-test comparison of DenseNet-121 [24] F1-scores under the baseline and proposed training settings.

Statistical significance was evaluated using Student’s *t*-test [32], while the magnitude of the observed improvement was measured using Cohen’s d [33], as illustrated in Figure 6. The analysis compared the F1-scores achieved by the proposed framework with those obtained from baseline training under the same dataset and evaluation protocol. The resulting *p*-value of 0.026 indicates statistical significance at the 95% confidence level. A Cohen’s d value of 0.96 indicates a large effect, based on the conventional thresholds of approximately 0.2, 0.5, and 0.8 for small, medium, and large effects, respectively. These findings demonstrate that the proposed framework provides a statistically significant and practically meaningful improvement in F1-score over the baseline.

## 5. Discussion

### 5.1. Information Fusion

Information fusion in the proposed framework refers to the balanced use of shape, texture, and pixel-intensity cues within the lung region. This fusion is performed only during the training phase, where the model is guided by mask-based perturbations and CAM-based attention correction to reduce excessive dependence on a single factor. As a result, the learned classifier carries this improved behavior into the testing phase without requiring any additional modules, functions, or structural changes. Therefore, the original backbone size and inference-time processing speed remain unchanged, while the classification performance and reasoning quality are improved. This training-only improvement without test-time overhead is a key idea of the proposed framework.

From a clinical perspective, reducing appearance bias can improve the reliability of AI-assisted chest X-ray interpretation by encouraging the classifier to rely on balanced and anatomically relevant evidence rather than a single dominant or irrelevant visual cue. Because AI-based classification can provide rapid predictions, the proposed framework may support clinicians in making faster and more reliable diagnostic decisions. The framework is intended as a decision-support tool rather than a replacement for clinical judgment.

The lung mask is not used to crop or replace the original input image; the classifier receives the complete chest X-ray, while the mask is used only to guide relatively greater attention toward the lung region and lower attention toward the surrounding background, which remains available and can still contribute to the final prediction. Current medical-image segmentation models already provide promising lung delineation performance, and continued improvements in segmentation are expected to further reduce errors caused by inaccurate mask boundaries.

### 5.2. Misclassified and Correctly Classified Cases

**Figure 7:**
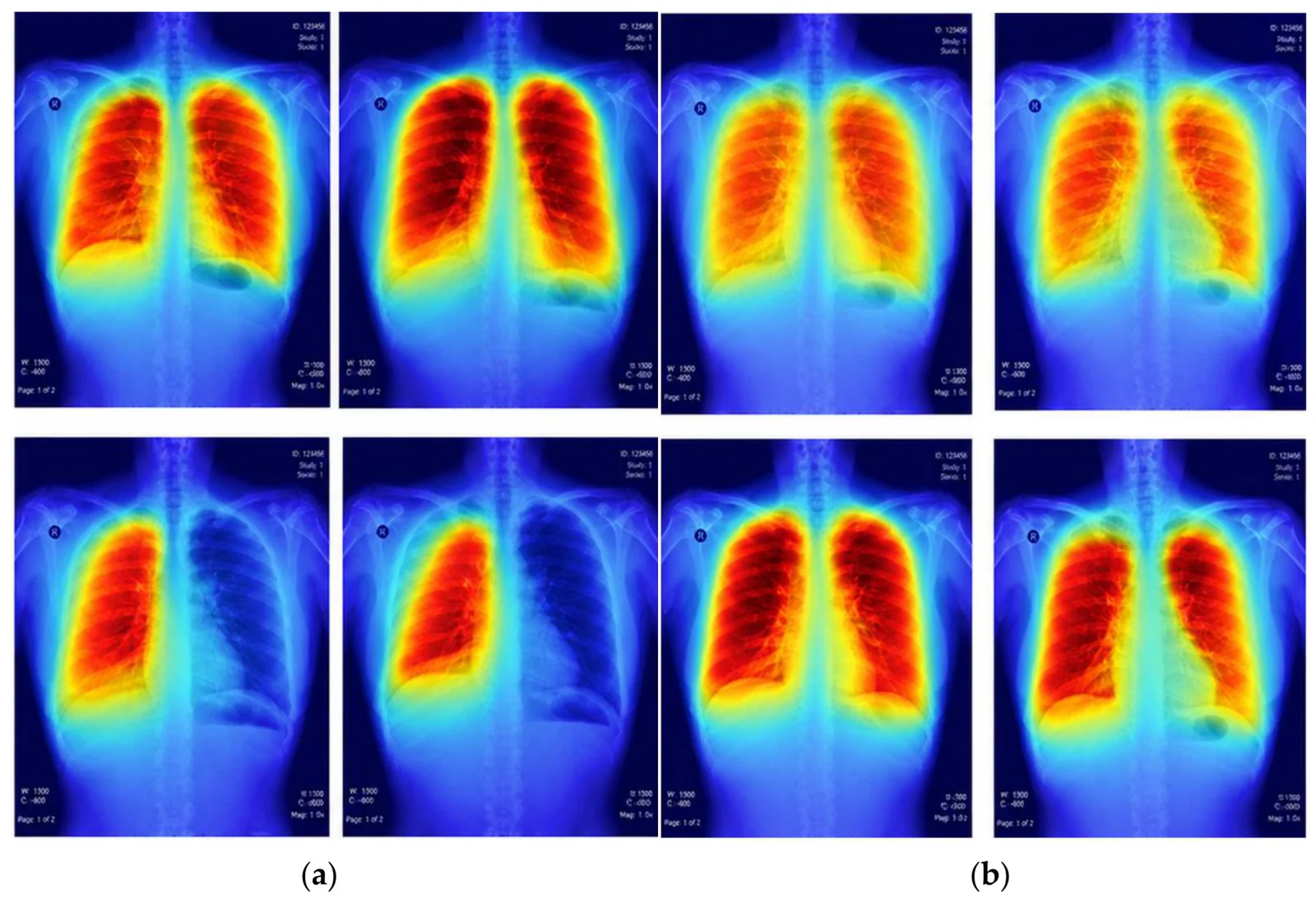
Representative misclassified and correctly classified examples. The upper and lower rows correspond to the baseline and proposed methods, respectively: (**a**) misclassified cases and (**b**) correctly classified cases.

Figure 7 presents representative correctly and incorrectly classified cases from DenseNet-121 trained with the proposed framework. For correctly classified samples, the method suppresses widely dispersed activation and directs the heatmap toward the lung fields, enabling the classifier to base its decision on more relevant regions. For misclassified samples, however, the activation may become overly confined to a limited lung area and fail to capture other informative features. Thus, although the framework generally promotes lung-focused attention, excessive localization can still contribute to incorrect predictions.

### 5.3. Limitations and Future Directions

Several limitations should be acknowledged. First, the available masks delineate the lungs rather than lesion-specific abnormal regions; therefore, improved attention alignment does not necessarily ensure that the model relies on class-relevant evidence. Second, the framework may occasionally concentrate attention too narrowly within a small lung area, potentially causing errors when informative features are subtle or distributed across multiple regions. Third, the training procedure requires both image-level annotations and paired lung masks, which increases annotation effort and computational cost. Finally, the experiments were conducted using only one publicly available dataset, and broader validation across additional medical imaging datasets is necessary.

Future research will investigate more precise or lesion-aware masks, stronger attention constraints to prevent excessively localized focus, and more stable training strategies on a variety of medical image databases [34] in addition to chest X-ray datasets. We also intend to study domain adaptation and assess the framework using additional public and clinical datasets to evaluate its robustness and generalizability across different hospitals, imaging devices, and acquisition conditions. In addition, we will investigate comparisons with expert-gaze datasets to assess whether model attention aligns with clinically relevant regions.

## 6. Conclusions

This study presented a pathology-aware, factor-aware training framework for chest X-ray classification. The proposed method addresses two major reasoning problems: appearance bias dominance, where the model becomes overly dependent on a single factor, and pathological attention drift, where the model focuses on frame or background rather than the lung region. To handle these issues, the framework uses CAM-based attention correction during training together with mask-guided perturbations that weaken texture, disturb shape, and reduce pixel intensity. In this way, the model is encouraged to learn more balanced and evidence-centered reasoning.

An important advantage of the proposed framework is that the additional guidance is applied only during the training phase. Therefore, the final trained model retains the same backbone structure, parameter size, and inference-time processing speed as the original baseline, while achieving improved reasoning behavior and better classification performance. The experimental results show that the proposed method improves not only the F1-score, but also the four proposed test-phase measures: reasoning stability, augmentation inconsistency, appearance bias dominance, and framing sensitivity. The proposed framework is bio-inspired because it encourages figure–ground separation, object-centered attention, and balanced use of complementary cues, thereby improving reasoning quality without changing the baseline model at test time.

Overall, the findings show that the proposed framework offers a practical means of enhancing both predictive performance and reasoning quality without altering the baseline model architecture. This training-only improvement without test-time overhead is the key contribution of the study. Future work will examine broader validation on additional medical datasets, more refined evidence masks, and stronger strategies for improving robustness under distribution shift.

## Figures and Tables

**Table 1:** Software and hardware used in our method.

Hardware		Software	
Hardware	Specification	Library	Version
GPU	Nvidia GeForce TITAN X (12 GB)	Python [16]	3.5.4
Memory	32 GB RAM	TensorFlow [17]	1.9.0
CPU	Intel(R) CoreTM i7-6700 CPU@3.40 GHz (8 CPUs)	OpenCV [18]	4.3.0
		Keras API [19]	2.1.6-tf

**Table 2:** Training hyperparameters and optimization settings.

Parameter	Classifier
Batch size	4
Learning rate	0.001
# Epochs	50
Loss	Categorical cross-entropy (CCE) [20]
Optimizer	Adaptive moment estimation (Adam) [21]

**Table 3:** Comparison of the baseline and proposed training methods.

Backbone	Training	F1	FS	AI	RS	ABD
EfficientNet-B0 [23]	Baseline	0.891	0.884	0.887	0.679	0.684
	Proposed	0.921	0.913	0.907	0.739	0.733
MobileNet-v1 [22]	Baseline	0.861	0.854	0.872	0.652	0.647
	Proposed	0.882	0.876	0.889	0.708	0.701
DenseNet-121 [24]	Baseline	0.899	0.904	0.894	0.694	0.698
	Proposed	0.931	0.936	0.914	0.756	0.749

**Table 4:** Comparison based on confusion matrices for DenseNet-121 (N, VP, LO, and CV denote Normal, Viral Pneumonia, Lung Opacity, and COVID).

	Predicted	Baseline				Proposed			
Actual		CV	LO	N	VP	CV	LO	N	VP
CV		425	48	0	27	467	23	0	10
LO		25	463	0	12	38	449	11	2
N		6	7	427	60	5	0	463	32
VP		2	0	17	481	2	2	12	484

**Table 5:** Comparison of the methods using class-wise PPV, TPR, and F1 for DenseNet-121.

Class	Baseline			Proposed		
	PPV	TPR	F1	PPV	TPR	F1
Normal	0.962	0.854	0.905	0.953	0.926	0.939
Viral Pneumonia	0.829	0.962	0.891	0.917	0.968	0.942
Lung Opacity	0.894	0.926	0.910	0.947	0.898	0.922
COVID	0.928	0.850	0.887	0.912	0.934	0.923

**Table 6:** Comparison of the baseline and proposed training method for different SOTA backbones.

Backbone	Training	F1	FS	AI	RS	ABD
ResNet18-SVM [25]	Baseline	0.889	0.891	0.877	0.664	0.664
	Proposed	0.921	0.925	0.901	0.729	0.729
Attention-CNN [26]	Baseline	0.839	0.844	0.854	0.631	0.631
	Proposed	0.864	0.869	0.879	0.691	0.691
ViT-ResNet Fusion [27]	Baseline	0.878	0.882	0.871	0.651	0.651
	Proposed	0.907	0.911	0.894	0.712	0.712
Fine-tuned Xception-XAI [28]	Baseline	0.869	0.873	0.878	0.678	0.678
	Proposed	0.893	0.897	0.902	0.741	0.741
Transfer-Learning Framework [29]	Baseline	0.862	0.866	0.882	0.691	0.691
	Proposed	0.887	0.891	0.905	0.753	0.753
Effective COVID-19 CNN [30]	Baseline	0.855	0.859	0.886	0.699	0.699
	Proposed	0.882	0.886	0.909	0.761	0.761

**Table 7:** Comparison of processing time and parameters (B and P denote the Baseline and Proposed methods, respectively).

Backbones	Method	Params (M)	FLOPs (G)	Inference Time per Image Unit as ms (fps)	
				Desktop	Jetson
DenseNet-121 [24]	B	8.0	2.83	24.42 (40.95)	62.75 (15.94)
	P				
EfficientNet-B0 [23]	B	5.3	0.39	11.96 (83.61)	42.86 (23.33)
	P				
MobileNet-v1 [22]	B	4.2	0.56	6.96 (143.68)	35.38 (28.26)
	P				
ResNet18-SVM [25]	B	11.7	1.82	17.93 (55.78)	52.50 (19.05)
	P				
Attention-CNN [26]	B	23.5	4.8	36.44 (27.44)	81.77 (12.23)
	P				
ViT-ResNet Fusion [27]	B	112.5	21.7	141.44 (7.07)	247.79 (4.04)
	P				
Fine-tuned Xception-XAI [28]	B	22.9	8.4	58.81 (17.00)	117.14 (8.54)
	P				
Transfer-Learning Framework [29]	B	5.3	0.39	11.96 (83.61)	42.86 (23.33)
	P				
Effective COVID-19 CNN [30]	B	3.8	0.08	7.12 (140.55)	35.40 (28.24)
	P				

## Data Availability

The original chest X-ray images and corresponding mask images used in this study are publicly available from the Kaggle dataset [15] described in the Dataset Description section. The source code for the proposed method is available on GitHub [2].
